# Sodium-pump gene-expression, protein abundance and enzyme activity in isolated nephron segments of the aging rat kidney

**DOI:** 10.14814/phy2.12369

**Published:** 2015-06-08

**Authors:** Pnina Scherzer, Anca Gal-Moscovici, David Sheikh-Hamad, Mordecai M Popovtzer

**Affiliations:** 1 Nephrology and Hypertension Services, Hadassah University HospitalJerusalem, Israel; 2 Nephrology Division, Department of Medicine, Baylor College of MedicineHouston, Texas; 3 South Arizona VA Health Care System and University of ArizonaTucson, Arizona

**Keywords:** Aldosterone, dissected tubules, Na^+^-K^+^- ATPase, senescent rats

## Abstract

Aging is associated with alteration in renal tubular functions, including sodium handling and concentrating ability. Na-K-ATPase plays a key role in driving tubular transport, and we hypothesized that decreased concentrating ability of the aging kidney is due in part to downregulation of Na-K-ATPase. In this study, we evaluated Na and K balance, aldosterone levels, and Na-K-ATPase gene expression, protein abundance, and activity in aging rat kidney. Na-K-ATPase activity (assayed microfluorometrically), mRNA (RT-PCR), and protein abundance (immunoblotting) were quantitated in the following isolated nephron segments: PCT, PST, MTAL, DCT, and CCD from 2, 8, 15, and 24 month-old-rats. In the course of aging, creatinine clearance decreased from 0.48 ± 0.02 mL/min/100 g BW to 0.28 ± 0.06 (*P* < 0.001) and aldosterone decreased from 23.6 ± 0.8 ng/dL to 13.2 ± 0.6 (*P* < 0.001). Serum Na^+^ and K^+^ increased by 4.0% and 22.5%, respectively. Na-K-ATPase activity, mRNA, and protein abundance of the *α*_1_ subunit displayed similar trends in all assayed segments; increasing in PCT and PST; decreasing in MTAL and DCT; increasing in CCD: in PCT they increased by 40%, 75%, and 250%, respectively; while in PST they increased by 80%, 50%, and 100%, respectively (*P* < 0.001). In MTAL they declined by 36%, 24%, and 34%, respectively, and in DCT by 38%, 59%, and 60%, respectively (*P* < 0.001). They were higher in CCD by 110%, 115%, and 246%, respectively (*P* < 0.001). Rats maintained Na/K balance; however with a steady state elevated serum K^+^. These results reveal quantitative changes in axial distribution of Na-K-ATPase at the level of gene expression, protein abundance, and activity in the nephrons of aging animals and may explain, in part, the pathophysiology of the senescent kidney.

## Introduction

Aging leads to profound anatomic and functional changes in a number of organs including the cardiovascular and respiratory systems. Much attention has been devoted to the effect of aging on the kidney (Bengele et al. 1981; Epstein 1979, 1996; Baylis and Corman 1998). Renal function undergoes significant changes during aging (Palmer and Levi 1996); these include progressive loss of nephron mass, hemodynamic changes, and abnormalities in electrolyte and water balance (Corman and Michel 1987; Geokas 1990). Impaired urinary concentrating ability of the aging kidney has been well documented in both humans (Lindeman et al. 1960; Rowe et al. 1976; Miller 2000) and experimental animals (Bengele et al. 1981). The functional changes in the senescent kidney that affect the tubules, refer primarily to defects in sodium handling (Macías Núñez et al. 1978; Luft et al. 1987). Abnormal tubular transport, like age-related decline in sodium hydrogen exchange (NHE3), sodium-dependent phosphate transport (NaPi-II) (Kinsella and Sacktor 1987; Sorribas et al. 1996), and impaired urinary concentration and dilution, have attracted much attention (O'neill and McLean 1992). Aging is also associated with reduced renin–angiotensin–aldosterone system, both in human and rat kidneys (Tsunoda et al. 1986; Jung et al. 1995). In this regard, AT_1_ mRNA expression is much lower in kidneys of 24-month-old rats compared with 3-month-old rats (Lu et al. 1996), and correlates well with decreased maximal binding of angiotensin II to its receptor in senescent animals. Decreased 1-alpha-hydroxylase activity with low circulating levels of l,25(OH)_2_D_3_ and abnormal calcium balance are other features of the aging kidney (Armbrecht et al. 1984).

The aging process results from cumulative effect of aging on individual cells. Moreover, abnormalities in cell–cell interactions are another pathophysiological feature of aging. Accordingly, there are aging-related changes in renal morphology which include vascular, glomerular, tubular, and interstitial tissues (Kaplan et al. 1975). There is a reduction in the number of nephrons, atrophy of tubular epithelium, tubular dilation, and increased thickness of basal membrane (Corman et al. 1985; Abrass et al. 1995).

Previous studies from our laboratory addressed changes in sodium pump in microdissected nephron segments from rats, using micromethods to assess changes in Na-K-ATPase activity in response to various pathophysiological conditions. These include, streptozotocin model of diabetes (Wald et al. 1986, 1993; Scherzer and Popovtzer 2002), acute tubular necrosis (Scherzer et al. 1990), heart failure (Wald et al. 1991), and others models (Scherzer et al. 1985, 1987; Wald et al. 1989; Rubinger et al. 1990). Because Na-K-ATPase is essential for tubular transport functions, we hypothesized that age-related changes in tubular transport and urine concentrating ability, correlate with changes in Na-K-ATPase abundance and activity; and in the current experiments, we examined this hypothesis. There are four catalytic *α* isoforms of Na-K-ATPase (*α*_1_, *α*_2_, *α*_3_, and *α*_4_) and 3 *β*-isoforms (*β*_1_, *β*_2_ and *β*_3_), expressed and regulated differentially, depending on the tissue type (Lingrel et al. 1990). The transporting enzyme Na-K-ATPase consists of a heterodimer of *α*-subunit and *β-*glycoprotein subunit (Sweadner 1989). The *α*-subunit is considered to be the catalytic and transporting subunit; it contains binding sites for ATP, Na^+^, K^+^, and cardiac glycosides (Lingrel et al. 1990). Thus, the *α*-subunit is responsible for ATP hydrolysis, which drives cation transport. The functional role of the glycosylated *β*-subunit is much less defined. It has been suggested that the *β*-subunit plays a role in the transfer of the *α*-subunit from the endoplasmic reticulum to the cell membrane, via the Golgi apparatus; thus, modulating the active enzyme (Ackermann and Geering 1990; McDonough et al. 1990). The *α*_1_ and *β*_1_ isoforms constitute the prevalent Na, K-ATPase isozyme in kidney; both *α*_1_ and *β*_1_ mRNAs are expressed at a similar level in each nephron segment, but their expression level varies along the nephron (Farman 1996).

We have examined the effect of aging on Na-K-ATPase gene expression (mRNA for *α*_1_ and *β*_1_ subunits), protein abundance, and activity along the nephron using dissected tubules. Compared with 2-month-old rats, the expression of *α*_1_ and *β*_1_ subunits and activity of the Na-K-ATPase were elevated in the proximal nephron [proximal convoluted tubule (PCT), and proximal straight tubule (PST)], lower in the medullary thick ascending limb (MTAL) and distal convoluted tubule (DCT), and higher in the cortical collecting duct (CCD). Lower activity of the Na-K-ATPase in MTAL was correlated with decreased urine osmolality as the rats aged; while lower Na-K-ATPase activity in the DCT was correlated with lower blood aldosterone levels with aging. Our data suggest that in spite of the decline in creatinine clearance (CrCl), rats maintained Na/K balance; however with a steady-state elevation in serum K^+^.

## Materials and Methods

Animal experiments were approved by the Institutional Animals Welfare Committee. Male rats of the Hebrew University strain Sabra were used in all studies. Rats aged 2 months served as the control group for adult rats aged 8, 15, and 24 months. Rats were fed standard Purina chow diet and drank tap water ad libitum. At the ages of 2, 8, 15, and 24 months, rats were housed in metabolic cages for several days for acclimatization. Food and water intake were measured for several days and the average of the measurements was recorded. Urine was collected for 24 h, blood was drawn for measurement of creatinine and aldosterone, and calculation of CrCl. Rats were killed by neck dislocation, the kidneys were removed immediately and the hilus was cut off and microdissected as previously described Scherzer et al. (1985). Isolated nephron segments were used to measure mRNA and protein abundance of the *α*_1_ and *β*_1_ subunits, and activity of Na-K-ATPase.

### Microdissection of tubule segments

Microdissection of isolated nephron segments was performed as described by Burg et al. (1966) and modified by Schmidt and Horster (1977)); the procedure was described in detail by Scherzer et al. (1985). Briefly, 0.5- to 1.0-mm-thick sagittal slices were cut and immediately immersed in dissection fluid [composition: 136 mmol/L NaCl, 3 mmol/L KCl, 1 mmol/L K_2_HPO_4_, 1.2 mmol/L MgSO_4_, 2 mmol/L CaCl_2_, 4 mmol/L sodium lactate, 1 mmol/L sodium citrate, 6 mmol/L l-alanine, and 5.5 mmol/L glucose) plus 0.6% collagenase (145 U/mg; Millipore, St. Louis, MO)]. The slices were incubated for 30 min in a shaking water bath at 37°C and aerated with 100% O_2_. The slices were then removed and rinsed twice in ice-cold dissection buffer. Cortical hemicircles, cut at the corticomedullary border, and medullary triangles, were used to obtain the appropriate segments. The slices were transferred to a petri dish with dissection buffer and inserted into a cooled Lucite chamber illuminated by a transmission dark-field source, as described by Schmidt and Horster (1977). The length of each segment was measured by an eyepiece micrometer. The segment was rinsed in fresh dissection buffer and transferred to a glass ampoule for analysis. The following cortical and medullary segments were dissected: PCT; PST; outer MTAL; DCT and CCD.

### Determination of ATPase activity

The method and apparatus for ATPase determination were previously described in detail (Schoner et al. 1967; Czaczkes et al. 1979; Scherzer et al. 1985). ATP hydrolysis is coupled to the transformation of phosphoenolpyruvate to pyruvate by pyruvate kinase. Pyruvate is reduced to lactate in the presence of lactic dehydrogenase. NADH acts as O_2_ acceptor and is oxidized to Nicotinamide adenine dinucleotide (NAD^+^). There is a stoichiometric relationship between the hydrolysis of ATP and the disappearance of NADH (by oxidation to NAD^+^). This disappearance is measured fluorometrically.

Na-K-ATPase is calculated as the difference between total ATPase and Mg^2+^-ATPase. The latter is determined by addition of ouabain (G-Strophantin) to a final concentration of 4 mmol/L. Na-K-ATPase activity is the mean of the difference of activities. About 3–5 pairs of nephron segments are dissected from each rat. Na-K-ATPase activity is compared among the groups; where 2-month-old rats, served as controls for 8-, 15-, and 24-month-old rats.

### RNA Isolation

Total RNA was extracted from pooled isolated nephron segments using a commercially available kit as per the manufacturer's instructions (RNeasy; Qiagen, Chatsworth, CA). After microdissection, pooled tubule segments (50–200 mm) were transferred into 5–10 *μ*L of microdissection solution added to 400 *μ*L of denaturing solution containing guanidinium Isothiocyanate. Final RNA pellets were resuspended in 30–60 *μ*L of diethylpyrocarbonate-treated water and quantified by absorbance at 260 nm.

### RT-PCR of *α*_1_ and *β*_1_ subunits of Na-K-ATPase

First-strand cDNA was synthesized from 0.2–0.5 *μ*g of total RNA. RNA samples were incubated for 1 h at 37°C with oligo[d(T)_21_] primer (Eistenberg Bros., Tel-Aviv, Israel), Maloney's murine leukemia virus reverse transcriptase (RT; 200 U/*μ*L; Promega, Madison, WI), and buffer containing 25 mmol/L MgCl_2_, 200 *μ*mol/L dNTPs, and RNase inhibitor (40 U/*μ*L) in a final reaction volume of 22 *μ*L. The reaction was stopped by incubation at 95°C for 10 min, and the cDNA was stored at −20°C until it was used. The primers for Na-K-ATPase subunits were selected by comparative nucleotide sequence analysis of published cDNA sequences by Schnieder et al. (1985) for *α*_1_ subunit and by Mercer et al. (1986) for *β*_1_ subunit. EcoRI and XbaI recognition sites were incorporated into the 5′ ends of the sense and antisense primers, respectively. Primers used for the *α*_1_ subunit were 5′- CCGGAATTCTGCCTTCCCCTACTCCCTTCTCATC-3′ (sense), and 5′-TGCTCTAGACTTCCCCGCTGTCGTCCCCGTCCAC-3′ (antisense), and the expected size of the polymerase chain reaction (PCR) product is 322 bp (nt 3207–3529; accession # info:ddbj-embl-genbank/NM_012504.1).

Primers used for the *β*_1_ subunit were 5′-GTTCGAATTCCCTCCGTCCTAATGACCCCAAGA-3′ (sense) and 5′-GCGGGATCCGACCAGAGCAGTTCCCCAGCCAGTC-3′ (antisense), and the expected size of the PCR product is 220 bp (nt 400–620; accession # NM_013113.2). The primers for the control gene glyceraldehydes-3-phosphate dehydrogenase (G3PDH) were purchased from Clontech; the expected length of the PCR product is 930 bp.

PCR was performed using a thermal cycler (Techne Progene; Oxford, UK) on 2–5 *μ*L aliquots of the first-strand cDNA. The PCR mixture contained 500 ng of each primer, 200 *μ*mol/L of each dNTP, 2.5 U of Ampli*Taq* polymerase (Promega), and reaction buffer containing 1.5 mmol/L MgCl_2_ in a final volume of 25 *μ*L. Amplification was performed as follows: 95°C for 2 min (initial denaturation), followed by 35 cycles: 95°C for 1 min, 55°C for 1 min, 72°C for 2 min; and a final extension at 72°C for 7 min. cDNA samples derived from four rats in each group were amplified simultaneously.

Negative controls included dissection medium subjected to RT-PCR, water subjected to PCR, and aliquots from each RNA sample without RT. All negative controls failed to generate PCR products.

### Analysis of PCR products

After amplification, 10 *μ*L of the PCR products were separated by electrophoresis on a 1.5% agarose gel containing ethidium bromide (0.5 *μ*g/mL; Sigma, Saint Louis, MO) in 40 mmol/L Tris acetate and 1 mmol/L ethylenediaminetetraacetic acid (EDTA) buffer. Ethidium bromide-stained bands of expected size were visualized under ultraviolet (UV) light. Band intensities of *α*_1_ and *β*_1_ subunits of Na^+^-K^+^-ATPase and the control gene G3PDH were quantified by densitometry (Multi-Analyst/PCT-Fluor-S Multi Imager System; Bio-Rad Laboratories, Hercules, CA). Band intensities of *α*_1_ and *β*_1_ subunits of Na-K-ATPase from each nephron segment were normalized to the corresponding bands/nephron segments, obtained from 2-month-old rats, which served as controls. Data are expressed as percent change in cDNA density of the different experimental groups compared with the control group.

### Southern blot analysis

To verify the molecular identities of G3PDH and the *α*_1_ and *β*_1_ subunits PCR products, we performed Southern blots. Gels were denatured, neutralized, and transferred to nylon membranes (Gene Screen, NEN Research Products, Boston, MA) according to the protocol of Manniatis et al. (1982) using 20× SSC (1× SSC = 0.15 mol/L NaCl and 0.015 mol/L sodium citrate, pH 7.0). The DNA was cross-linked to nylon membranes by UV light (Ultraviolet Transilluminator, Cole-Parmer, Chicago, IL). Blots were hybridized for 16–20 h with ^32^P-labeled cDNA fragments corresponding to the *α*_1_ and *β*_1_ subunits of Na^+^-K^+^-ATPase under stringent conditions. Radioactive probe was prepared using a DNA labeling kit (Rediprime; Amersham/VWR, Arlington Heights, IL). Hybridizations were performed using radiolabeled cDNA probe for the *α*_1_ subunit of Na-K-ATPase (nt 3060–3636, accession # M14137.1) and an *Eco*RI fragment of the *β*_1_ subunit of Na-K-ATPase (nt 1–850, accession # M14137.1) (Schnieder et al. 1985). Membranes were washed and autoradiographed. Bound cDNA probes were stripped by boiling in 1× SSC for 2 min, and the membranes were rehybridized with ^32^P-labeled G3PDH cDNA probe. Bands were quantified by phosphorimaging (Fuji × model BHS 1000) and normalized to G3PDH cDNA. Data represent the mean and ±SEM of four independent determinations.

### Measurement of protein abundance of Na-K-ATPase *α*_1_ and *β*_1_ subunits

Pooled tubules of defined length (at least 100 mm/assay) were lysed in Tri-Reagent™ (Sigma) and protein was isolated as per the manufacturer's instructions (Chomczynski 1993). Samples were resolved on sodium dodecyl sulfate polyacrylamide gel electrophoresis (SDS-PAGE; 10% acrylamide) and blotted onto nitrocellulose membrane as described (Shyjan and Levenson 1989). Blots were stained with Coomassie Brilliant Blue R-20 (Sigma) to verify protein transfer.

The blots were reacted with the following antibodies: rabbit anti-rat Na-K-ATPase *α*_1_ (UPState Biotechnology, Lake Placid, NY) at a dilution of 1:3000; rabbit anti-Na-K-ATPase *β*_1_ (UPState Biotechnology) at a dilution of 1:7000. The antibody–antigen complexes were visualized by incubation with goat anti-rabbit serum (dilution of 1:10,000; Bio Rad Laboratories), followed by enhanced chemiluminescence and autoradiography. Densitometry was performed using phosphor imager. Equal protein loading was verified using Coomassie Brilliant Blue R-20 staining.

### Biochemical assays

Blood and urine samples were analyzed for creatinine, sodium, and potassium. Urine samples were also analyzed for protein content and osmolality. Measurement of aldosterone level in the blood was also carried out.

Creatinine concentration in the blood and urine was determined (for CrCl calculation) by an automated picric acid method using autoanalyzer of Cobas Mira (Hoffmann-La Roche and Limited Diagnotstica, Basel, Switzerland). Direct measurement of sodium and potassium was carried out on undiluted samples using ion selective electrodes (ISE; Cobras Mira autonanalyzer). Urine protein was detected by Pyrogallol Red direct colorimetric method, also using autoanalyzer (Cobas Mira). Urine osmolality was measured using Fiske osmometer (Fiske Associates, Norwood, MA). Plasma aldosterone levels were determined by radioimmunoassay (Coat-A-Count; Diagnostic Products Corporation, Los Angeles, CA). Data are presented as mean ± SEM for the ages 8, 15, and 24 months, using 2-month-old rats as control.

### Statistical analysis

Values are expressed as means ± SEM. Analysis of variance was performed for statistical evaluation between the groups. Results of individual groups were compared using nonpaired Student's *t*-test with a modified level of significance according to Godfery's method (Godfrey 1985).

## Results

### Kidney function

Metabolic data and measurements of kidney function are listed in Tables1 and 2. An increase in body weight was observed with aging. This change was progressive and significant when compared with 2-month-old rats (Table1). CrCl is shown, both as an absolute value and factored for body weight. In terms of absolute value, CrCl displayed the following pattern: CrCl increased from 1.39 ± 0.05 mL/min in 2 month-old rats to 2.45 ± 0.16 in 8-month-old rats (*P* < 0.001); at 15 and 24 months, CrCl did not differ significantly from that observed in 8-month-old rats, but was higher than observed in 2-month-old rats (*P* < 0.05). When normalized to body weight, CrCl displayed the following pattern: CrCl did not differ in 8 month--old rats compared to 2-month-old rats; however, CrCl was lower in 15- and 24-month-old rats, compared to 2- and 8-month-old rats (*P* < 0.005). Urine osmolality in 24-month-old rats was lower by 60% compared to 2-month-old rats (*P* < 0.001). This decrease was accompanied by a commensurable increase in urine volume, from 8.3 ± 0.6 mL/24 h in 2-month-old - to 17.0 ± 1.8 mL/24 h in 24-month-old rats (*P* < 0.001; Table1).

**Table 1:** Metabolic data of 2-, 8-, 15-, and 24-month-old rats

Months	2 (*n* = 9)	8 ( *n* = 10)	15 ( *n* = 10)	24 (*n* = 10)
Weight g	289 ± 13	495 ± 34*	637 ± 28*	677 ± 16*
CrCl mL/min	1.39 ± 0.12	2.45 ± 0.20*	1.71 ± 0.08	1.88 ± 0.15
CrCl mL/min/100 g B.W	0.48 ± 0.02	0.495 ± 0.04	0.267 ± 0.03*	0.277 ± 0.06*
Urine volume mL/24 h	8.3 ± 0.60	9.5 ± 1.60	14 ± 3.20	17 ± 1.80*
Urine Osmolality mOsmol/kg H_2_O	3540 ± 425	2809 ± 361*	1613 ± 136*	1410 ± 176*

* *P* < 0.05 versus 2 months.

**Table 2:** Blood Na, K, and aldosterone levels; and urinary excretion of Na and K of rats with age; 2, 8, 15, and 24 months

Months	2 (*n* = 7)	8 (*n* = 9)	15 (*n* = 9)	24 (*n* = 9)
Blood Na ± mEq/L	141.31 ± 1.18	145.22 ± 1.56	145.81 ± 2.73	147.20 ± 1.22*
Blood K ± mEq/L	4.22 ± 0.13	5.46 ± 0.37*	5.42 ± 0.29*	5.45 ± 0.26*
UNaV *μ*Eq/min	0.56 ± 0.13	0.71 ± 0.06*	0.75 ± 0.05*	1.49 ± 0.14*
UKV *μ*Eq/min	1.31 ± 0.11	1.68 ± 0.15	2.00 ± 0.31*	3.03 ± 0.02*
Blood Aldosterone ng%	23.6 ± 0.82	13.2 ± 1.63*	15.7 ± 1.43*	13.7 ± 1.63*

* *P* < 0.05 versus 2 months.

As shown in Table2, urine sodium excretion increased starting from age 8 months, through 24 months. An increase in potassium excretion was also observed in 8-month-old rats; however it was significant only at the age of 15 months. Protein excretion increased from 6.1 ± 0.15 mg/24 h at age 2 months, to 57 ± 6.7 mg/24 h at 24 months, a ninefold increase (*P* < 0.001).There was an increase in blood K^+^ level, from 4.22 ± 0.13 at 2 months to 5.46 ± 0.37 at 8 months; K^+^ remained at the same levels through 24 months of age (*P* < 0.05). Blood Na level rose from 141.3 ± 1.18 mEq/L at 2 months to 147.2 ± 1.22 mEq/L at 24 months (*P* < 0.005). Aldosterone levels decreased from 23.6 ± 0.82 ng/dL at 2 months to 13.2 ± 1.62 ng/dL at 8 months (*P* < 0.001), and remained low until the age of 24 months (Table2).

### The effects of aging on gene expression of *α*_1_ and *β*_1_ subunits of Na-K-ATPase, protein abundance, and activity along the proximal nephron

#### Gene expression

**Figure 1:**
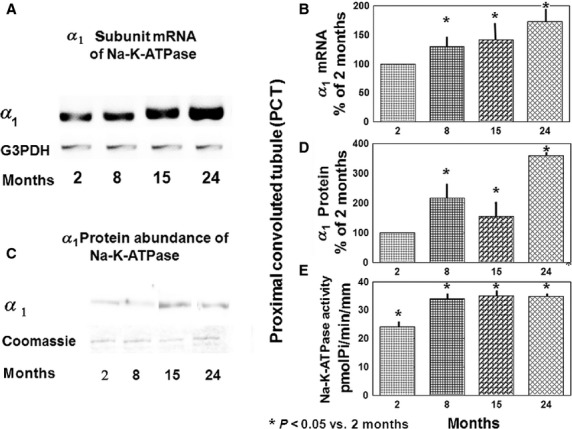
Na-K-ATPase gene expression, protein abundance, and activity in PCT from 2-, 8-, 15-, and 24-month-old rats. (A) Products of representative RT-PCR for *α*_1_ subunit mRNA of Na-K-ATPase and control mRNA (G3PDH) run on ethidium bromide-stained gel of 2, 8, 15, and 24-month- old rats. (B) Densitometry of *α*_1_ of Na-K-ATPase mRNA products (as shown in A) in the isolated PCT of 2, 8, 15, and 24 months old rats. Data represent the mean and ±SEM from four independent determinations; *P < 0.05 versus 2 months old rats. (C) Representative immunoblots of protein abundance of *α*_1_ subunit of Na-K-ATPase in the PCT of 2, 8, 15, and 24-month-old rats. Coomassie Blue staining verified equal amounts (30 *μ*g) of proteins loaded on the gel. (D) Abundance of *α*_1_ subunit based on densitometry measurements of immunoblotting; data represent the mean and ±SEM from four independent determinations. (E) Na-K-ATPase activity in the PCT from 2-, 8-, 15-, and 24-month-old rats. Na-K-ATPase activity in PCT from 10 pairs of segments for each age group; *P < 0.005 versus 2 months.

Using RT-PCR on microdissected proximal nephron segments, and compared with 2-month-old rats, we observed an increase in the *α*_1_ subunit (322 bp product), while the *β*_1_ subunit (220 bp product) was decreased by 24 months. Negative controls demonstrated no contamination in the reaction mixture, and Southern blots with *α*_1_ and *β*_1_ mRNA and G3PDH-specific probes confirmed the identity of the PCR products (data not shown). Expression of the control gene G3PDH (930 bp) was unchanged in PCTs from age 2, through 24 months of age. Figure1A depicts representative gel of RT-PCR products of *α*_1_ mRNA subunit in PCT, while Fig.1B summarizes the densitometry data for the *α*_1_ subunit mRNA in isolated PCT of 2-, 8-, 15-, and 24-month-old rats. The expression of the *α*_1_ subunit mRNA of Na-K-ATPase in the PCT increased by 30%, 42%, and 73% at ages 8, 15, and 24 months, respectively (*P* < 0.001 vs. 2 months old rats); while in PST we observed an increase of 49% and 27% in 15- and 24-month-old rats, respectively (Table3). Expression of *β*_1_ mRNA of Na-K-ATPase along the nephron is shown in Table4; the changes were variable and inconsistent.

**Table 3:** mRNA of α_1_ subunit Na-K-ATPase along the nephron of the aging rats

Segment → Month	PCT	PST	MTAL	DCT	CCD
2 (*n* = 4)	100 ± 3	100 ± 4	100 ± 3	100 ± 5	100 ± 1
8 (*n* = 4)	130 ± 2*	100 ± 5	98 ± 5	41 ± 3*	200 ± 10*
15 (*n* = 4)	142 ± 1.5*	149 ± 3*	79 ± 1*	45 ± 4*	158 ± 9*
24 (*n* = 4)	173 ± 6*	127 ± 2*	70 ± 2*	46 ± 1*	215 ± 13*

* *P* < 0.05 versus 2 months.

**Table 4:** mRNA of *β*_1_ subunit of Na-K-ATPase along the aging rat nephron

Segment → Months	PCT	PST	MTAL	DCT	CCD
2 (*n* = 4)	100 ± 2	100 ± 5	100 ± 4	100 ± 3	100 ± 3
8 (*n* = 4)	100 ± 5	100 ± 5	152 ± 12*	50 ± 4*	200 ± 16*
15 (*n* = 4)	100 ± 4	33 ± 2*	164 ± 13*	52 ± 3*	91 ± 9
24 (*n* = 4)	81 ± 4*	38 ± 5	87 ± 5*	40 ± 5*	43 ± 6*

* *P* < 0.05 versus 2 months.

### Protein abundance

Typical results of the immunoblotting for *α*_1_ (112kD) and *β*_1_ (52kD) subunits of Na-K-ATPase in the isolated PCT from 2-, 8-, 15-, and 24-month-old rats are shown in Fig.1C. Protein abundance of *α*_1_ subunit of Na-K-ATPase increased in PCT of 8, 15 and 24-month-old rats by 110%, 56%, and 260%, respectively (*P* < 0.005; Fig.1D). Protein abundance of *β*_1_ subunit of Na-K-ATPase in PCT increased 35% only at 24 months of age (*P* < 0.005; Table6).

In PST, the abundance of *α*_1_ subunit of Na-K-ATPase followed the same pattern as that in PCT (Table5); that is, an increase of 50%, 45%, and 100% in 8-, 15-, and 24-month-old rats, respectively. Abundance of the *β*_1_ subunit of Na-K-ATPase in PST was similar to that in PCT, increasing only by 45% at 24-month-old rats (*P* < 0.001; Table6).

**Table 5:** α_1_ subunit protein abundance of Na-K-ATPase along the nephron in aging rats

Segment → Month	PCT	PST	MTAL	DCT	CCD
2 (*n* = 4)	100 ± 3	100 ± 4	100 ± 3	100 ± 5	100 ± 1
8 (*n* = 4)	210 ± 12*	150 ± 5*	108 ± 7	100 ± 5	150 ± 11*
15 (*n* = 4)	156 ± 5*	145 ± 3*	76 ± 3*	42 ± 4*	213 ± 10*
24 (*n* = 4)	360 ± 16*	200 ± 9*	77 ± 4*	60 ± 3*	346 ± 17*

* *P* < 0.05 versus 2 months.

**Table 6:** The *β*_1_ subunit abundance of Na-K-ATPase along the nephron in aging rats

Segment → Months	PCT	PST	MTAL	DCT	CCD
2 (*n* = 4)	100 ± 1	100 ± 4	100 ± 1	100 ± 0.5	100 ± 1
8 (*n* = 4)	100 ± 7	110 ± 6	88 ± 5*	56 ± 4*	80 ± 4*
15 (*n* = 4)	106 ± 4	110 ± 7	76 ± 5*	30 ± 3*	249 ± 15*
24 (*n* = 4)	135 ± 6*	145 ± 4	37 ± 6*	13 ± 2*	221 ± 16*

* *P* < 0.05 versus 2 months.

### Na-K-ATPase activity

Typical Na-K-ATPase activity in the proximal nephron is shown in Fig.1E. In PCT, the activity increased 31% at 8 months of age and persisted through 24 months of age; while in PST, the activity increased by 83% and 56% at the ages of 15 and 24 months, respectively (Table7).

**Table 7:** Na-K-ATPase activity along the nephron in aging rats (µMole Pi/min/mm tubule)

Segment → month	PCT	PST	MTAL	DCT	CCD
2 (*n* = 10)	24.3 ± 2.8	22.5 ± 1.8	41 ± 0.5	42 ± 1	15.2 ± 0.3
8 (*n* = 10)	33.1 ± 0.8*	22.4 ± 1.4	40 ± 0.75	31 ± 0.8*	25 ± 0.4*
15 (*n* = 10)	35.2 ± 0.7*	41.2 ± 3*	35 ± 0.3*	28 ± 1.2*	20 ± 0.5*
24 (*n* = 10)	34.5 ± 0.6*	35.3 ± 1.5*	30 ± 0.4*	30 ± 0.7*	20.8 ± 0.7*

* *P* < 0.05 versus 2 months.

### Gene expression and protein abundance of *α*_1_ and *β*_1_ subunits, and activity of Na-K-ATPase along the distal nephron of the aging rat

#### Gene expression

**Figure 2:**
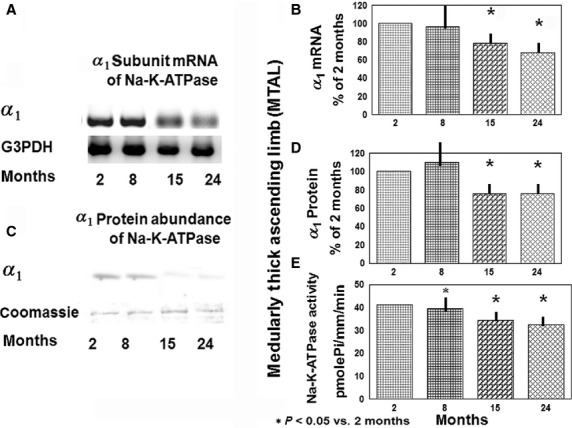
Na-K-ATPase gene expression, protein abundance, and activity in MTAL from 2-, 8-, 15-, and 24-month-old rats. (A) Representative gels for *α*_1_ subunit mRNA of Na-K-ATPase and control gene (G3PDH) mRNA on ethidium bromide-stained gels. (B) Densitometry of *α*_1_ mRNA of Na-K-ATPase levels in isolated MTAL of 2-, 8-, 15-, and 24-month-old rats (shown in A). Data represent the mean and ± SEM from four independent determinations; *P < 0.05 versus 2-month-old rats. (C) Representative immunoblots show protein abundance of *α*_1_ subunit of Na-K-ATPase in the MTAL of 2, 8, 15, and 24-month- old rats. Coomassie Blue staining verified equal amounts (30 *μ*g) of proteins loaded on the gel. (D) Data represent the mean and ±SEM from four independent determinations. (E) Na-K-ATPase activity in the MTAL from 2-, 8-, 15-, and 24-month-old rats. Na-K-ATPase activity in MTAL from 10 pairs of segments for each group at each age; *P < 0.05 versus 2 months.

Figure2A depicts representative gels of RT-PCR products of *α*_1_ mRNA in MTAL; Fig.2B shows densitometry of the corresponding RT-PCR products. A decrease in *α*_1_ mRNA of the enzyme by 21% and 30% in the MTAL was observed in 15- and 24- month- old rats, respectively. A fall of 59% in the *α*_1_ mRNA of Na-K-ATPase in 8-month-old rats is seen in the DCT (Table3), which persists through 24 months of age. Conversely, in CCD the *α*_1_ mRNA of Na-K-ATPase increased by 100%, 58%, and 115% in 8-, 15-, and 24-month-old rats, respectively (*P* < 0.001; Table3).

Changes in *β*_1_ mRNA of Na-K-ATPase in the MTAL, DCT and CCD are shown in Table4. In DCT the *β*_1_ subunit mRNA decreased by 50%, 48% and 60% in 8-, 15-, and 24-month-old rats, respectively, mirroring the fall in the mRNA of *α*_1_ subunit. While in the MTAL and CCD, the *β*_1_ mRNA subunit behaves differently from that of *α*_1_ mRNA, increasing in MTAL at ages 8 and 15 months, but only at age 8 months of age in CCD; while the *α*_1_ mRNA decreased slightly in MTAL at ages 15 and 24 months, and doubled in the CCD at ages 8, 15, and 24 months.

### Protein abundance

**Figure 3:**
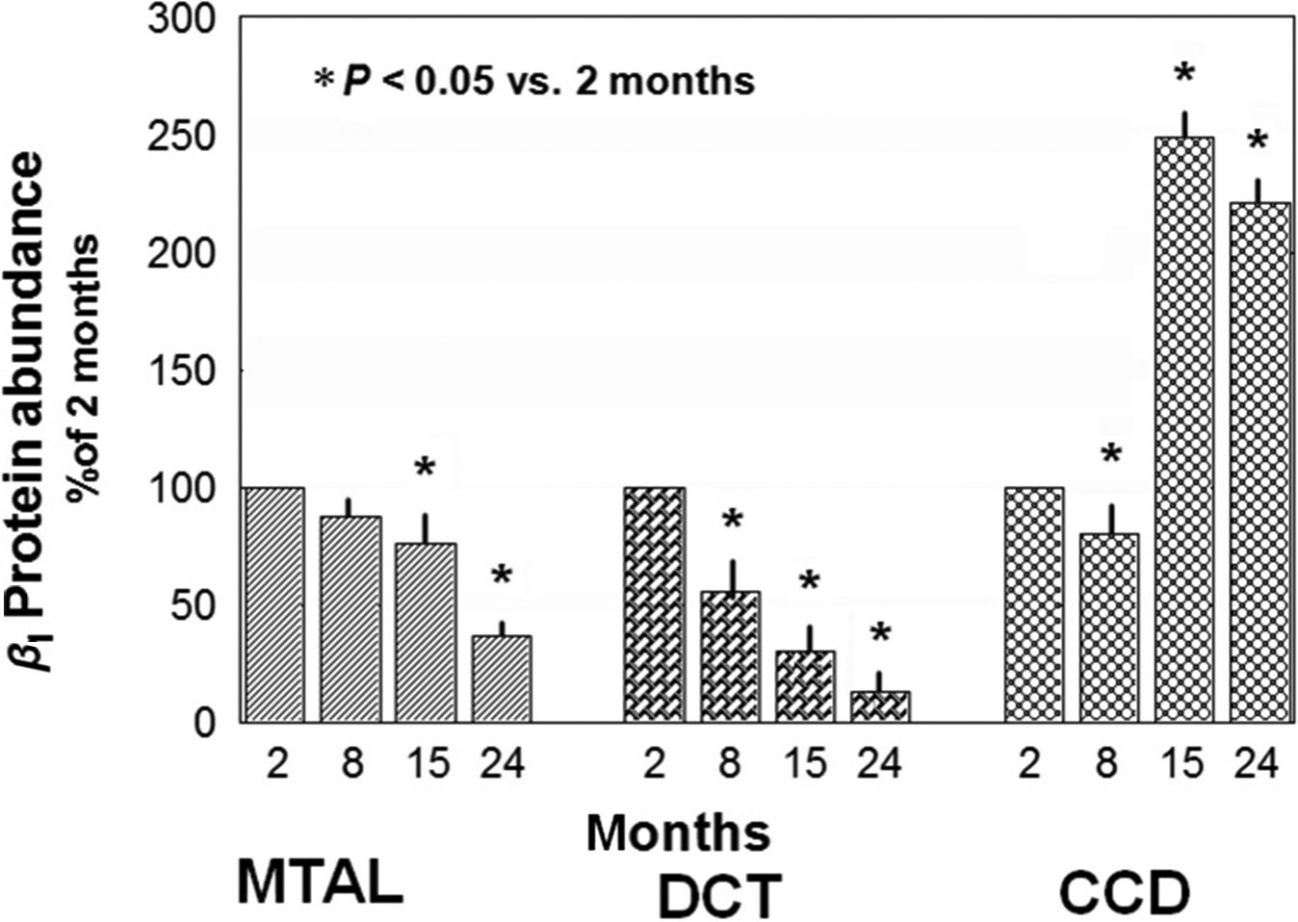
Changes in the abundance of *β*_1_ subunit of Na-K-ATPase along the distal nephron in 2-, 8-, 15-, and 24-month-old rats; *P* < 0.05 versus 2-month-old rats.

Figure2C depicts representative immunoblots of *α*_1_ subunit in MTAL. Densitometry measurements of the immunoblots for MTAL, DCT, and CCD are depicted in Table5. The abundance of *α*_1_ Na-K-ATPase in the MTAL decreased by 34% and 33% in 15- and 24-month-old rats, respectively (Fig.2D). Similarly, it decreased by 58% and 40% in DCT of 15- and 24-month-old rats, respectively. While in CCD the abundance of *α*_1_ subunit rose gradually and peaked at 24 months to 346%. Changes in the abundance of *β*_1_ subunit of Na-K-ATPase in the distal nephron parallel those of the *α*_1_ subunit; that is, progressively with aging, there is a significant decrease in the abundance of *β*_1_ subunit in MTAL and DCT and an increase in CCD (Fig.3).

### Na-K-ATPase activity

Na-K-ATPase activity in MTAL is depicted in Fig.2E, while the activity in DCT and CCD is depicted in Table7. In the MTAL and DCT there is a decline in enzyme activity during aging; in MTAL it decreases 15% at age 15 months and 27% at age 24 months (*P* < 0.001). However, enzyme activity increased in CCD at 8, 15, and 24 months by 66%, 33%, and 36% respectively (*P* < 0.005).

### Correlation between *α*_1_ subunit mRNA of Na-K-ATPase and aging along the nephron

**Figure 4:**
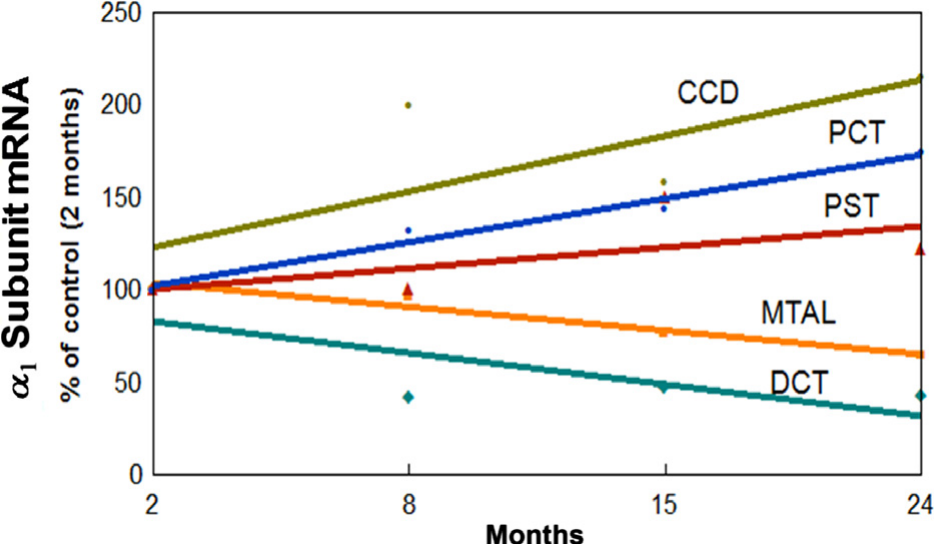
Correlation between *α*_1_ subunit mRNA of Na-K-ATPase along the nephron and aging. The correlation is given as percentage of levels in 2- month-old rats, assigned a value of 100%. Data represent the mean and ±SEM from four independent determinations.

The correlation between *α*_1_ subunit mRNA and aging along the nephron is shown in Fig.4, and is depicted as percentage of values in 2-month-old rats. There is a linear increase in the *α*_1_ mRNA of Na-K-ATPase during aging in PCT, PST, and CCD (*r* = 0.98, *P* < 0.001 for PCT; *r* = 0.53, *P* < 0.005 for PST; and *r* = 0.76, *P* < 0.005 for CCD), while a negative linear correlation is observed in the MTAL and DCT (*r* = −0.97, *P* < 0.001 for MTAL; *r* = −0.75, *P* < 0.001 for DCT).

### Correlation between *α*_1_ subunit protein abundance of Na-K-ATPase along the nephron with aging

**Figure 5:**
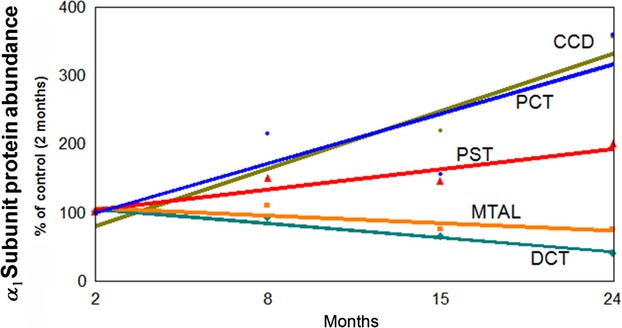
Correlation between *α*_1_ subunit protein abundance of Na-K-ATPase along the nephron and aging. The changes in *α*_1_ subunit abundance are given as percentage of levels in 2-month-old rats, assigned a value of 100%. Data represent the mean and ± SEM from four independent determinations.

Changes in protein abundance of the *α*_1_ subunit along the nephron were compared to values obtained from 2-month-old rats, and correlated with age (Fig.5). In PCT, PST, and CCD, abundance of the *α*_1_ subunit rose in a linear fashion with age (*r* = 0.83, *P* < 0.025 for PCT; *r* = 0.93, *P* < 0.05 for PST; *r* = 0.97, *P* < 0.05 for CCD), while in the MTAL and DCT there is a linear decrease (*r* = −0.89, *P* < 0.01 for MTAL; *r* = −0.97, *P* < 0.025 for DCT).

### Correlation between Na-K-ATPase activity along the nephron with aging

**Figure 6:**
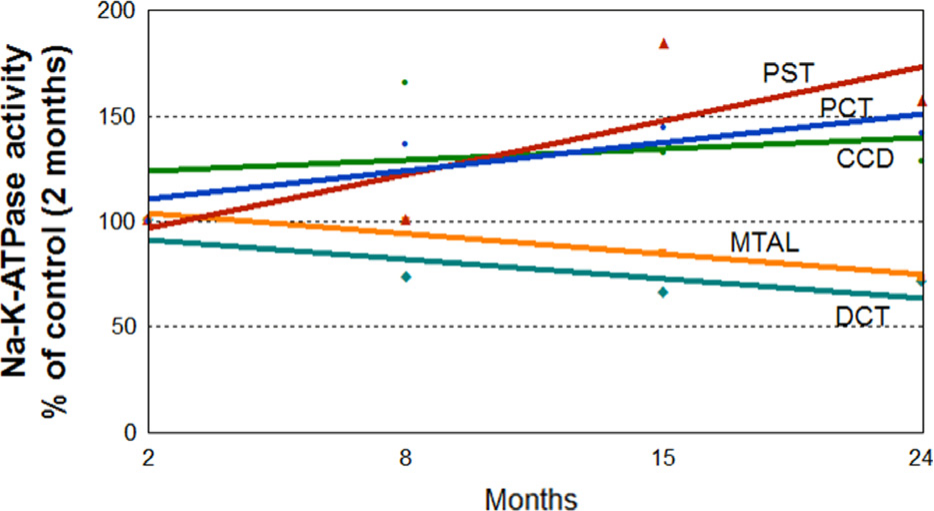
The relationship between Na-K-ATPase activity along the nephron, and aging. The changes in the Na-K-ATPase activity in the different nephron segments compared to 2-month-old rats, assigned a value of 100%. Values are from 10 pairs of segments for each age group.

Changes in Na-K-ATPase activity along the nephron with aging were compared to values obtained from 2-month-old rats (Fig.6). In the PCT, PST, and CCD there was positive and linear correlation with aging (*r* = 0.96, *P* < 0.001 for PCT; *r* = 0.53, *P* < 0.05 for PST; and *r* = 0.69, *P* < 0.005 for CCD), while an inverse relationship between aging and Na-K-ATPase activity is observed in the MTAL and DCT (*r* = −0.94, *P* < 0.001 for MTAL; and *r* = −0.75, *P* < 0.001 for DCT).

### Correlation between Na-K-ATPase activity in MTAL and urine osmolality with aging

We observed an inverse and linear correlation between Na-K-ATPase activity in the MTAL and urine osmolality during aging (*r* = 0.97, *P* < 0.01); that is, decreased enzyme activity in MTAL is correlated with decreased urine concentration. Since solute transport by the MTAL is critical for establishing the medullary concentration gradient, the data suggest that decreased Na-K-ATPase activity in the MTAL may underlie the defect in urine concentrating ability in the senescent kidney.

### Correlation between aldosterone levels and Na-K-ATPase activity in the DCT

We observe an inverse and linear correlation between aldosterone levels and Na-K-ATPase activity in the DCT (*r* = −0.82, *P* < 0.02).

## Discussion

The focus of present study was to explore the hypothesis that changes in Na-K-ATPase expression and activity along the nephron may underlie the decline in transport function and diminished concentrating ability of the aging kidney. To that end, we examined gene expression, protein abundance, and activity of the Na-K-ATPase along the nephron in the aging rat kidney. Previous studies addressed this issue by analyzing renal tissue slices and/or homogenates employing differential centrifugation to isolate-specific anatomic regions of the nephron. We have adopted a more direct approach, by microscopic dissection and isolation of specific segments of the nephron. The following tubular segments were dissected and isolated: PCT, PST, MTAL, DCT, and CCD. This method yielded new information which may bear on the pathophysiology of the aging kidney.

The highlights of the present study are the findings that aging is associated with progressive downregulation of the Na-K-ATPase at the level of gene expression, protein abundance, and activity in the MTAL and DCT. In contrast, the PCT, PST, and CCD, show upregulation of Na-K-ATPase gene expression, protein abundance, and enzyme activity. While, the *β*_1_ subunit follows the same pattern displayed by the *α*_1_ subunit in MTAL, DCT, and CCD, it does so to a lesser extent in the PCT and PST; being variable and less consistent. These differences between the *α*_1_ and *β*_1_ subunits are not surprising as the genes for *α*_1_ and *β*_1_ subunit are located on different chromosomes, and as pointed out by McDonough et al. (1994), expression of the two subunits of Na-K-ATPase is not always correlated and one subunit may be limiting for the constitution of the holoenzyme. Accordingly, the expression of one subunit may be sufficient (if limiting) or not (if not limiting) to induce functional consequences.

The progressive downregulation of Na-K-ATPase gene expression, protein abundance, and activity in MTAL and DCT with aging is not clearly defined, but thus may have potential consequences on the function of the senescent kidney. The Na^+^ pump is the major driving force for vectorial transepithelial Na^+^ reabsorption in polarized epithelia. Since the Na-K-2Cl cotransporter is the main gate for Na^+^ entry into the MTAL, its downregulation suggests possibly reduced Na+ presentation to the Na^+^ pump with a consequent decline in pump activity. Thus, the observed decline in Na^+^ pump in MTAL might reflect diminished Na^+^ delivery from the proximal nephron. This possibility is supported by the observed increase in Na^+^ pump in PCT and PST segments consistent with increased proximal Na^+^ reabsorption leading to reduced distal delivery of Na^+^ to MTAL. Our findings are in agreement with previously published data in this regard. Bengele et al. (1983) demonstrated reduced Na-K-ATPase activity (38%) in the renal medulla of old rats compared with young rats. While Tian et al. (2006) demonstrated decreased abundance of bumetanide-sensitive Na-K-2CL cotransporter in the outer medulla of aging rat kidneys. Likewise, previous studies have demonstrated reduced vasopressin-dependent reabsorption of sodium by the TAL of Henle's loop in senescent mice kidneys (Di Stefano et al. 1991). Our observations and those by others, lend support to the notion of reduced sodium reabsorption by the MTAL of the aging kidney.

Gottschalk and Mylle (1959) showed sodium reabsorption from the ascending limb of the loop of Henle is the “single effect” that drives the countercurrent exchanger establishing and maintaining an osmotic gradient in the medulla of the kidney. Thus, sodium reabsorption by MTAL is key for maintenance of high corticomedullary gradient for water reabsorption and urine concentration. The present experiments demonstrated an age-related progressive decline in urinary osmolarity, from 3540 in 2-month-old rats to 1410 mOsm/kg H_2_O in 24-month-old rats. Furthermore, the close correlation between MTAL Na-K-ATPase activity and urine osmolality during aging support the notion that a fall in Na^+^ uptake in MTAL may be the underlying mechanism for impaired urinary concentration and dilution in the senescent kidney.

Clinical observations on renal concentrating capacity are in accordance with experimental studies by us and others. Impaired renal concentrating ability has been clearly demonstrated in the elderly in comparison to the young (Meyer 1989; Geokas 1990). Water diuresis studies in elderly subjects demonstrated decreased NaCl transport in the ascending loop of Henle (Macías Núñez et al. 1978). Thus, the clinical findings are consistent with the experimental results indicating reduced Na^+^ reabsorption in the ascending limb of Henle's loop. A defect in solute transport in Henle's loop could account for both; impaired maximum urine concentration and maximum urine dilution that characterize the aging kidney. Polyuria associated with impaired sensation of thirst during dehydration that often occurs in the elderly may increase the risk of hyperosmolality and hypernatremia which may become serious life-threatening complications.

The age-related progressive decline in Na-K-ATPase gene expression, protein abundance, and activity in DCT is poorly understood. In view of the reduced Na^+^ uptake in MTAL, one would predict an increased Na^+^ delivery to DCT, prompting increased Na^+^ uptake by DCT with stimulated Na-K-ATPase activity; however, the opposite was observed. Downregulation of the Na^+^ pump in DCT could not be attributed with certainty to reduced apical thiazide-sensitive Na^+^Cl^-^ cotransporter, as recent study showed no decrease or even an increase in the abundance of this electroneutral transporter in senescent rat kidney (Tian et al. 2006). The above study, however, was conducted on kidney homogenates or by immunohistochemistry and not in isolated renal tubules. Furthermore changes in abundance do not necessarily reflect similar changes in activity.

The present study demonstrates an age-related fall in plasma aldosterone level, from 23.6 to 13.2 ng/dL. A similar fall in plasma aldosterone in aging rats was reported by Eiam-Ong and Sabatini (1999), but not by Tian et al. (2006). The latter authors, however, found a decrease in renin activity. An age-related fall in plasma aldosterone was also reported in men (Weidmann et al. 1975). A 30% to 50% decrease in supine plasma aldosterone levels was reported in elderly versus younger subjects; this difference was exaggerated with upright posture (Flood et al. 1967; Hegstad et al. 1983). This age-related aldosterone deficiency is more likely related to a renin–angiotensin deficiency than to an intrinsic adrenal defect. Three- to fivefold elevation in basal ANP levels has been found in healthy elderly compared with young adults (Haller et al. 1987; Ohashi et al. 1987; McKnight et al. 1989). ANP suppresses the renin–angiotensin–aldosterone system, thus it may contribute to the low levels of aldosterone in the elderly.

We have also demonstrated a close correlation between plasma aldosterone levels and Na-K-ATPase activity in DCT. This is of interest as aldosterone has been shown to stimulate Na^+^ transport in this segment (Velázquez et al. 1996; Kim et al. 1998). Furthermore, aldosterone has also been shown to increase the abundance of the thiazide-sensitive Na^+^-Cl^-^ cotransporter in DCT. These observations raise the possibility that the decline in Na-K-ATPase activity in DCT is caused by diminished aldosterone levels in plasma. DCT plays an important role in salt and water balance, as it continues the dilution process started by the ascending limb of Henle. Interference with the diluting function may lead to hyponatremia which is more common in elderly patients treated with thiazide diuretics.

Our study demonstrates a robust upregulation of the Na^+^ pump in CCD. This finding is surprising in view of the age-related dramatic fall in plasma aldosterone level. Our previous experiments in isolated tubular segments demonstrated close correlation between aldosterone levels and Na-K-ATPase activity in various experimental settings (Wald et al. 1993). Aldosterone plays a major role in the regulation of electrolyte transport in CCD, partly by stimulating Na-K-ATPase which is the key enzyme that energizes Na^+^ reabsorption via amiloride-sensitive epithelial Na^+^ channel (ENaC) and facilitates K^+^ secretion across K^+^ channels. The mechanism of the observed aldosterone-independent rise in Na-K-ATPase in CCD remains enigmatic. Increased delivery of Na^+^ from TAL and DCT to CCD is a possible mechanism. In addition, elevated serum K^+^ concentration as observed in our study may stimulate Na-K-ATPase activity as well.

Our experiments in rats demonstrate a significant rise in serum K^+^ concentration that paralleled the decrease in plasma aldosterone levels during the course of aging (Table2). In this regard, Doucet and Katz (1980) have shown that chronic K^+^ loading in rats is associated with stimulation of Na-K-ATPase in the collecting duct. They also demonstrated that upregulation of Na-K-ATPase was observed in the CCD of adrenalectomized rabbits, and was not inhibited by the aldosterone antagonist spironolactone in the CCD of mice (Garg and Narang 1985, 1987). These observations suggest that stimulation of Na-K-ATPase by K^+^ in the CCD may be aldosterone-independent, at least partially. Consistently, recent experimental studies demonstrated dissociation between aldosterone levels and Na-K-ATPase activity in CCD. Lourdel et al. (2005) have shown that the presence of aldosterone and activation of epithelial Na^+^ channel in CCD are not required for sodium retention in Puromycin-induced nephrosis (PAN) in rats. These investigators demonstrated increased Na-K-ATPase in CCD in adrenalectomized corticoid-clamped PAN rats. The animals avidly retained Na^+^ despite decreased number and activity of ENaC. This finding was interpreted by the investigators as indicating that induction of Na-K-ATPase was sufficient to increase Na^+^ retention. While the ENaC number and activity were dependent on aldosterone, induction of Na-K-ATPase was aldosterone-independent.

Thus, our data suggest that in certain circumstances Na-K-ATPase activity in the CCD may change in a fashion independent of alterations in aldosterone levels. Of note, during sodium retention in a model of experimental cirrhosis in rats, Kim et al. (2005) demonstrated downregulation of type 2 11-betahydroxysteroid dehydrogenase in CCD. This allowed increased signaling through mineralocorticoid receptor independent of circulating aldosterone level. Thus, examination of type 2 11-betahydroxysteroid dehydrogenase in CCD may provide some insight into the regulation of Na-K-ATPase in CCD during aging.

The fall in aldosterone by 44% raised the risk of hyperkalemia. In fact, concomitant with the fall in aldosterone, the mean serum K^+^ concentration rose from 4.4 to 5.5 mEq/L (20%). This increase in K^+^ level persisted from 2 to 24 months, and it is possible that the aldosterone-independent increase in Na^+^ pump helped preserve K^+^ balance and prevent hyperkalemia during the course of aging.

In summary, our study is the first to examine gene expression, protein abundance, and activity of the Na-K-ATPase in isolated nephron segments of the aging kidney, and shows altered axial expression and activity of the Na-K-ATPase, in the nephrons of aging animals as compared with those in young animals. The changes we observe in Na-K-ATPase along the nephron during the aging may shed some light on the mechanism(s) that underlie the pathophysiology of the senescent kidney. Additional studies are needed to better define the complex and poorly understood mechanisms that account for the altered physiology of senescent kidneys.
